# A Novel Constraint for Thermodynamically Designing DNA Sequences

**DOI:** 10.1371/journal.pone.0072180

**Published:** 2013-08-28

**Authors:** Qiang Zhang, Bin Wang, Xiaopeng Wei, Changjun Zhou

**Affiliations:** 1 Key Laboratory of Advanced Design and Intelligent Computing (Dalian University), Ministry of Education, Dalian, China; 2 School of Mechanical and Engineering, Dalian University of Technology, Dalian, China; UC Davis School of Medicine, United States of America

## Abstract

Biotechnological and biomolecular advances have introduced novel uses for DNA such as DNA computing, storage, and encryption. For these applications, DNA sequence design requires maximal desired (and minimal undesired) hybridizations, which are the product of a single new DNA strand from 2 single DNA strands. Here, we propose a novel constraint to design DNA sequences based on thermodynamic properties. Existing constraints for DNA design are based on the Hamming distance, a constraint that does not address the thermodynamic properties of the DNA sequence. Using a unique, improved genetic algorithm, we designed DNA sequence sets which satisfy different distance constraints and employ a free energy gap based on a minimum free energy (MFE) to gauge DNA sequences based on set thermodynamic properties. When compared to the best constraints of the Hamming distance, our method yielded better thermodynamic qualities. We then used our improved genetic algorithm to obtain lower-bound DNA sequence sets. Here, we discuss the effects of novel constraint parameters on the free energy gap.

## Introduction

More than half a century has passed since the double helix configuration of DNA was identified [1]. Presently, such knowledge about DNA contributes to virtually every area of science, including the use of DNA as a computational tool [2]. The hybridization reaction between 2 DNA sequences is important for advanced DNA applications because its efficiency and accuracy directly influence application reliability; however, false hybridization is an unavoidable artifact of combining DNA strands due to biotechnical limitations. False hybridizations occur as false positives and false negatives [3], [4]. A false positive hybridization is a new duplex formed by mismatched single DNA sequences, due to a lack of single-strand similarities. A false negative hybridization involves matching DNA sequences that do not hybridize at all due to biochemical errors [5], [6]. DNA sequence design is critical to many biotechnological applications. DNA microarrays rely on accurate DNA design of probes that are immobilized on a surface and bind specifically to complementary targets in a complex mixture [7], [8]. Designing DNA sequences which satisfy some constraints could reduce false positives and improve hybridization uncertainty and inaccuracy between probes and their complementary targets. Designed DNA sequences should satisfy single or combinational constraints to ensure DNA sequence quality and permit the shortest DNA sequence to code for each informational unit required. Accurate DNA production also reduces false hybridizations and improves accuracy. The goal of DNA sequence design is to find the maximal number of designs that satisfy single or combinatorial constraints as well as the smallest design that satisfies these constraints.

We propose a novel distance criterion for designing DNA sequences. Using the novel free energy gap constraint, we designed DNA with better thermodynamic properties. Then, an improved genetic algorithm was used to search the lower bounds of DNA sequence sets that satisfy the novel and combinatorial constraints. Finally, we describe the relationship between the thermodynamic properties of DNA sequence sets and the parameters of novel constraints.

## Methods

### Free Energy Gap Criterion

Biotechnical limitations contribute to DNA hybridization uncertainties and inaccuracies which limit available DNA-based applications. To improve hybridization between two DNA molecules, investigators have explored DNA sequence thermodynamic properties to control the MFE and melting temperature. As a criterion for measuring thermodynamic properties of DNA sequences, the MFE of a sequence or sequences is the minimum value among free energies of all possible conformations of a sequence (s) [9]. Here, we report our efforts using the online freeware PairFold to predict the MFE of two interacting DNA molecules and gauge the quality of DNA sequence sets by using the free energy gap δ.

A DNA sequence
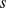
is a string composed of alphabet Σ = {A,C,G,T}. Δ*G*(*u,v*) denotes the value of MFE between DNA sequences *u* and *v*, which is calculated by PairFold [9]. In addition, *s′* denotes the Watson-Crick reverse-complement sequence of DNA sequence *s*. S is the set of DNA sequences *s*, S′ is the set of *s*′. To calculate the free energy gap δ, definitions are stated as follows:

(1) Sequence-Sequence Constraint: for all pairs of *u_i_*, *v_j_* in S,

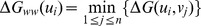
(1)(2) Sequence-Complement Constraint: for all pairs of *u_i_* in S, *v_j_* in S′, and *i* ≠ *j*,

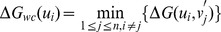
(2)(3) Complement-Complement Constraint: for the pairs of *u_i_*, *v_j_* in S′,

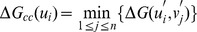
(3)(4) Sequence-Self-Complement Constraint: for all pairs of *u_i_* in S, *u_i_* in S′, and *u* = (*u*′)′,

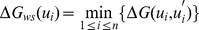
(4)(5) Free energy gap: denoted by δ. For two DNA sequences *u* and *v*,
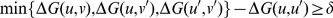
(5)where 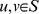 and 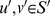. In general, a larger δ represents a larger gap between the free energy of desired and undesired hybridizations, and thus a better set (DNA sequence set quality) [7], [10]. We used the free energy gap to gauge the quality of DNA sequence sets constrained by the Hamming distance and the novel constraint, namely the longest aligning common substring distance constraint (LACS). By comparing the free energy gap, we measured the influence of different distance constraints on DNA sequence designs.

### The Hamming Distance Constraint

The Hamming distance constraint is frequently used to reduce DNA sequence similarity for DNA-based applications and mainly includes the word-word Hamming distance constraint (WWH) and the word-complement Hamming distance (WCH).

Garzon first proposed the definition problem of designing DNA sequences for DNA computing [11] as follows: in the alphabet Σ = {A,C,G,T}, there exists a set *S* with length *n* and size of |S| = 4*^n^*. A subset 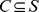 and let *u*, *v* any two codes in the *C* satisfy:
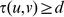
(6)
*d* is a positive integer, τ is the constraint criteria (or criterion) for DNA sequences, such as the Hamming distance criterion.

#### Word-word Hamming distance (WWH)

Word-word Hamming distance constraint: for the DNA sequences *u*,*v* with given length *n* (written from the 5′ to the 3′ end), *H*(*u*,*v*) denotes the Hamming distance between *u* and *v*. *WWH*(*u_i_*) denotes the minimal *H*(*u_i_*,*v_j_*) in all DNA sequences and should not be less than parameter *d*,
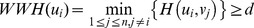
(7)


#### Word-complement Hamming distance (WCH)

Word-complement Hamming distance: for DNA sequences *u*,*v*′ with given length *n* (written from the 5′ to the 3′ end), *H*(*u*,*v*′) denotes the Hamming distance between *u* and *v*′. *WCH*(*u_i_*) denotes the minimal *H*(*u_i_*,*v′_j_*) in all DNA sequences and should not be less than parameter *d*, i.e.,
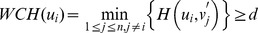
(8)


#### GC content constraint

The GC content constraint approximates the thermodynamic properties of DNA sequences and is combined with the distance constraint. A fixed percentage of nucleotides within each DNA sequence is *G* or *C*. Using this constraint, we assume this percentage is (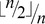)%.

### The Novel Distance Constraint

We propose the novel LACS distance constraint for the design of DNA sequences. DNA sequences that satisfy the novel constraint show reduced similarity and exhibit better thermodynamic properties than sequences constrained by Hamming distance.

#### Similarity

The LACS distance denotes [*l*,*k*] = *LACS*(*A*,*B*); *l* is the length of the longest consecutive common substring between *A* and *B*, *k* is the number of positions (excluding the longest consecutive common substring) at which the corresponding symbols are the same while aligning the location of the longest common substring between *A* and *B*.

**Figure 1:**
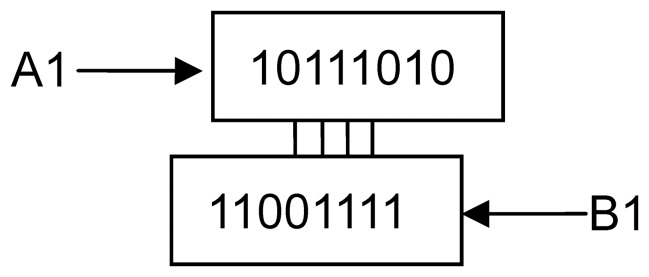
Example of *A_1_* and *B_1_*.

**Figure 2:**
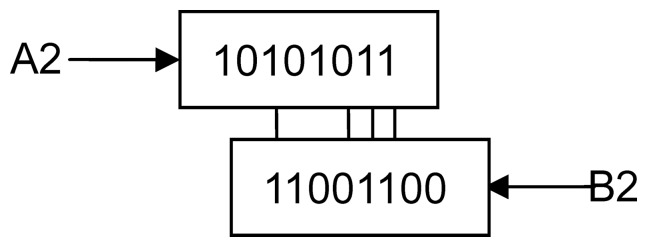
Example of *A_2_* and *B_2_*.

For example, strings *A*_1_ = 10111010, string *B*_1_ = 11001111, *A*_2_ = 10101011, *B*_2_ = 11001100, [4,0] = *LACS*(*A*_1_,*B*_1_), [3], [1] = *LACS*(*A*_2_,*B*_2_). First, *A*_1_ and *B*_1_ align the location of the longest common substring as in Fig. 1, in which we find that the longest consecutive common substring is 0111, so *l* = 4. At the same, other aligned sequences are not equal, then *k* = 0. For *A*_2_ and *B_2_*, they are aligned as the location of the longest common substring as in Fig. 2, in which we find that the longest consecutive common substring is 011, so *l* = 3. After alignment using the longest consecutive common substring, the third subsequence of *A*_2_ is equal to the first subsequence of *B_2_*, the *k* = 1.

In this paper, *n* denotes the length of the DNA sequence. According to the definition of Hamming distance denoted by *d_H_* for designing DNA sequences, we define the LACS distance *d*_L_, *d_L_* = *n* – *l* – *k.* Generally speaking, the smaller the values of *l* and *k* and the larger the value of *d*_L_, the smaller the similarity between 2 strings.

#### Thermodynamic property

MFE is the minimum free energy of all possible structures and the most effective approach to control for unexpected secondary DNA sequence structures. The algorithm of PairFold and the standard thermodynamic parameters for DNA molecule are based on the nearest-neighbor thermodynamic model [9]; therefore, we employed the LACS distance constraint to approximate the MFE between 2 DNA sequences (not necessarily matching each other) that could form secondary structures.

#### Word-word LACS distance (WWL)

Word-word LACS distance constraint: for the DNA sequences *u*,*v* with given length *n* (written from the 5′ to the 3′ end), *WWL*(*u_i_*) denotes the maximal *LACS*(*u_i_*,*v_j_*) in all DNA sequences, where the values of *l* and *k* should not be more than parameters *t_l_* and *t_k_*, respectively.
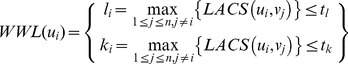
(9)


#### Word-complement LACS distance (WCL)

Word-complement LACS distance: for the DNA sequences *u*,*v*′ with given length *n* (written from the 5′ to the 3′ end), *WCL*(*u_i_*) denotes the maximal *LACS*(*u_i_*,*v′_j_*) in all DNA sequences, where the values of *l* and *k* should not be more than parameters *t_l_* and *t_k_*, respectively.
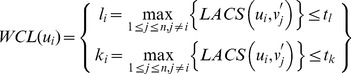
(10)


Here, we employed the improved genetic algorithm to design DNA sequence sets which satisfy the combinatorial constraints based on the different distance, and gauged the quality of the sets using the free energy gap calculated by PairFold [19]. Comparing free energy gaps can verify which distance constraint is better for DNA design. To improve the quality of DNA sequence design, the number of the LACS is equal to 1 in each pair of DNA sequences.

### Algorithm Design

Genetic algorithms (GAs) are adaptive heuristic search algorithms based on evolutionary concepts of natural selection and genetics. An improved genetic algorithm to design DNA sequence sets based on the LACS distance constraints could enhance global search capabilities of a traditional genetic algorithm based on DNA sequence set characteristics. Improvements include initializing algorithm populations with the evenly distributed method. This enhances multiformity of populations based on a global field. According to the number of populations, the populations are evenly distributed in the value scope by the evenly distributed method. Randomly re-initializing the populations when they satisfy certain conditions would overcome premature convergence. Population re-initialization occurs once because increased time decreases the convergence of the algorithm. In the mutation process, we adjusted the probability of a mutation operator with a dynamic method. The traditional genetic algorithm adopts unique values to process the mutation operation, which could reduce convergence. The optimization problem is defined by the problem of maximum value, and we employ an average weight to manage the evaluation function. We denote fitness function *f*(*i*):
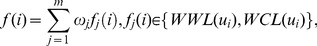
(11)where *ω_j_* = 1 is the weight of each constraint, *m* is the number of constraints, and *f_j_*(*i*) are the selected constraints.

**Figure 3:**
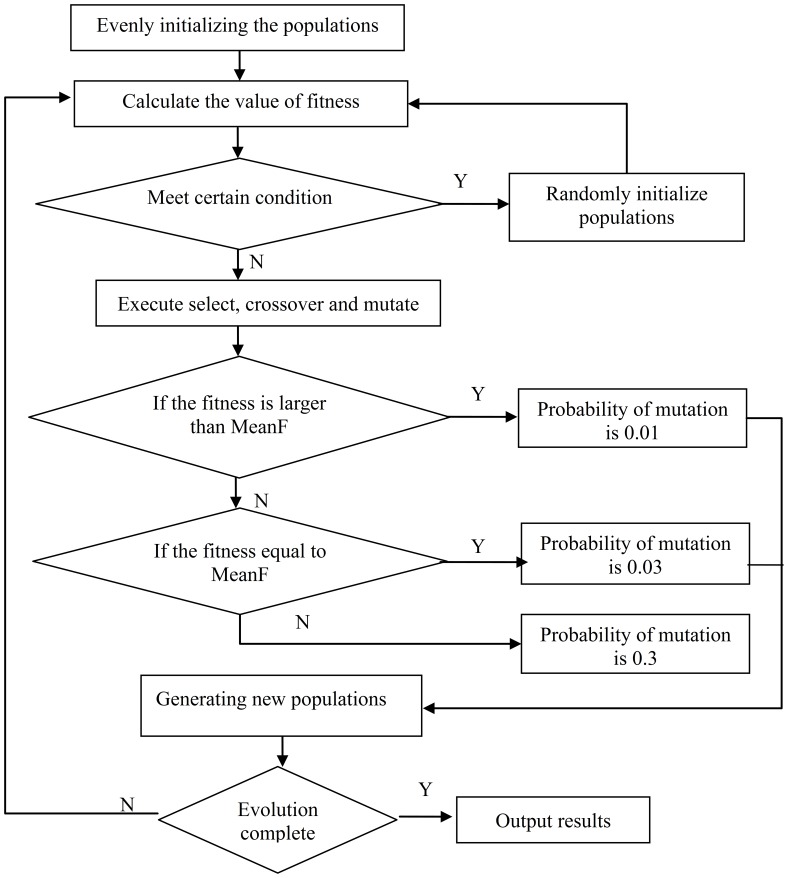
Algorithm process flow.

The algorithm initializes DNA sequences with an evenly distributed method, selecting sequences which satisfy the constraint (or constraints), generating new DNA sequences by selection, crossover, and the mutation operator, and finally yielding the desired DNA sequence sets. Figure 3 illustrates the process flow.

The steps for designing DNA sequence sets with the improved genetic algorithm are as follows:

Step 1: Set parameters and initialize the population with an evenly distributed method.

Step 2: Calculate the value of fitness function. We employed the MeanF to denote the mean of the fitness function. If 
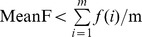
, then randomly re-initializing the populations.

Step 3: Generate the next generation population by selection, crossover, and mutation. The algorithm uses random tournament selection and the three-point crossover strategy. The size of the tournament is 2 and the number of repetitions is equal to 10% of the total population in the random tournament selection. In the mutation process, if fitness is larger than MeanF, its probability of mutation is 0.01, and if it is equal to MeanF, its probability of mutation is 0.03. Otherwise, its probability of mutation is 0.3. This process yields dynamic adjustment of the probability. If the generation is less than 200, the algorithm proceeds to step 2; if not, the algorithm moves to step 4.

Step 4: End and output results.

Our algorithms were successful with many different combinatorial constraints. Our results were better than those described in previous reports [12], [13]. Thus, our algorithm is sufficient to design DNA sequence sets which satisfy the LACS distance constraint.

## Results

The parameters of the improved genetic algorithm in our example are as follows: population size 1000, crossover 0.45, initial probability of a mutation is 0.01. To control the run time of the algorithm, the number of generations is 200. We used the PairFold package [9] to calculate the MFE of 2 DNA sequences. According to recent research, no statistically significant differences exist among free energy approximations in 4 publicly available and widely used programs [8]. The temperature in the algorithm is 37°C. To increase the reliability of our experimental results, we performed 50 experiments for every value and reported the mean of these experiments. In the tables, *d* is the distance based on the Hamming, LACS, or both constraints, and *n* is the length of the DNA sequence. Blank cells contain the ‘–’ symbol.

### Comparing the Free Energy Gaps

In Tables 1 and 2, *d* is the distance based on the Hamming and LACS constraints, for which *d* = *d_H_* = *d_L_*. Data in Table 1 are the free energy gaps of DNA sequence sets which satisfy the WWL and WCL combinatorial constraints. Parenthetical data are the free energy gaps that satisfy the WWH and WCH combinatorial constraints in Table 1. Table 2 data are the free energy gaps that satisfy the WWL, WCL, and GC content combinatorial constraints. Data in parentheses are the free energy gaps that satisfy the WWH, WCH, and GC content combinatorial constraints in Table 2.

**Table 1:** Data with two combinatorial constraints.

n\d	4	5	6	7	8
4	1.408 (0.526)	–	–	–	–
5	1.148 (0.574)	2.144 (1.060)	–	–	–
6	1.172 (0.018)	2.348 (0.454)	2.968 (1.600)	–	–
7	1.380 (−0.018)	2.170 (0.780)	3.958 (2.142)	4.180 (2.558)	–
8	1.582 (−0.246)	2.128 (0.084)	3.044 (0.840)	4.858 (2.268)	5.162 (3.324)

**Table 2:** Data with three combinatorial constraints.

n\d	4	5	6	7	8
4	1.542 (0.716)	–	–	–	–
5	1.938 (0.456)	2.454 (1.236)	–	–	–
6	1.726 (0.496)	3.040 (1.608)	3.470 (2.044)	–	–
7	1.944 (0.282)	2.692 (1.246)	3.888 (2.410)	4.362 (2.586)	–
8	2.588 (0.264)	3.246 (0.030)	3.982 (0.652)	5.118 (2.042)	5.594 (3.102)

Tables 1 and 2 depict data based on the Hamming distance constraint [13], for which we used the same experimental parameters and algorithms, including the experiment run times. In order to verify the influence of the LACS constraint in the design of DNA sequences, we compared the free energy gaps based on the Hamming distance and LACS distance constraints, while having the same length of DNA sequences and same distance constraint.

A comparison of the free energy gaps based on different distance constraints (Tables 1, 2) suggest the LACS distance constraint is better than the Hamming distance constraint for designing DNA sequences. The data in the tables suggest the quality of DNA sequence sets constrained by LACS distance constraint is significantly better after adding the GC content constraint. Thus, the novel constraints express thermodynamic properties more relevant to the MFE. Comparisons with the Hamming distance constraint do not account for values of *l* and *k*.

### Lower Bounds of DNA Sequence Sets

Table 3 depicts the lower bounds of the designed DNA sequence sets that satisfy different combinatorial constraints. *d* is the distance based on the LACS constraint, where *d* = *d_L_*. Data shown are DNA sequence sets that satisfy the WWL and WCL combinatorial constraints and parenthetical data represent sizes that satisfy the WWL, WCL, and GC content combinatorial constraints. DNA sequence set sizes differ for each unique value *l* or *k*, while DNA sequence lengths are constant (*d_L_*). Maximal sizes are depicted in Table 3.

**Table 3:** Size values to satisfy different combinatorial constraints.

n\d	4	5	6	7	8
4	2 (1)	–	–	–	–
5	3 (2)	2 (1)	–	–	–
6	5 (5)	3 (2)	1 (1)	–	–
7	6 (5)	5 (4)	2 (2)	2 (1)	–
8	11 (9)	6 (6)	4 (4)	2 (2)	1 (1)

Table 3 also depicts the lower bounds of DNA sequence sets which satisfy different combinatorial constraints based on the LACS criterion. The data suggest that DNA sequence set sizes would be reduced by adding the GC content constraint. Free energy gap values increased with increases in LACS distance, whereas DNA sequence set sizes decreased, similar to the Hamming distance [13].

### The Relations between the Parameters of LACS

When the DNA sequence sets are the same length and have the same distance constraints, their free energy gaps often differ. To investigate the influence of different values of *l* and *k* on the free energy gap value in same-length DNA sequences with the same distance constraints, we used DNA sequence sets with *n* = 8 as the analytic example and free energy gaps as the criterion. Tables 4 and 5 depict data for the free energy gaps constrained by the combinatorial constraints. Parenthetical data are values of *k*. Tables 4 and 5 also demonstrate that free energy gaps increase with increasing values of LACS distance (See Tables 1 and 2 for similar characteristics). Also, free energy gaps decrease with increasing values of *k*, keeping *l* constant. Also, free energy gaps decrease with increasing the values of *l*, keeping *k* constant. Finally, the maximum free energy gap was best estimated using a maximum value of *l*, the LACS distance.

**Table 4:** Data without GC content.

d\l	2	3	4	5	6	7
1	–	–	0.286 (3)	0.244 (2)	0.404 (1)	0.366 (0)
2	–	–	0.642 (2)	0.860 (1)	1.208 (0)	–
3	1.528 (3)	0.866 (2)	1.322 (1)	1.942 (0)	–	–
4	1.736 (2)	1.582 (1)	2.388 (0)	–	–	–
5	2.128 (1)	2.582 (0)	–	–	–	–

**Table 5:** Data with GC content.

d\l	2	3	4	5	6	7
1	–	–	0.542 (3)	0.432 (2)	0.646 (1)	0.742 (0)
2	–	–	0.624 (2)	1.448 (1)	1.522 (0)	–
3	2.512 (3)	2.512 (2)	1.992 (1)	2.192 (0)	–	–
4	2.770 (2)	2.588 (1)	2.800 (0)	–	–	–
5	3.250 (1)	3.246 (0)	–	–	–	–

## Discussions

The distance constraint (or the similarity constraint) is the chief method for designing DNA sequences. Constraints such as the Hamming distance are used to reduce the similarity of DNA sequences used in hybridization reactions by describing the minimum number of substitutions required to change one DNA stand into the other. Simple mathematical formulae are used to confirm the similarity of a pair of DNA sequences, helping to reduce the likelihood of false positives; however, this technique does not accurately address the thermodynamic properties of DNA sequences, even when accounting for GC content. Addressing thermodynamic constraints for DNA design would increase sequence accuracy more than present design strategies based on distance constraints. Baum proposed the existence of DNA sequence similarity [5] and suggested constraints that could be used in DNA sequence design. He also described maximum DNA sequence sets that would satisfy these constraints. Deaton proposed that DNA sequence design should be combined with biochemical techniques and reported coding reliability problems when information theory was used alone [6]. Deaton suggested an evolutionary genetic algorithm to design DNA sequences. In contrast, Hartemink proposed DNA design based on distance constraints (such as the Hamming distance) and the free-energy criterion [14]. Improving on these discoveries, Zhang used an improved genetic algorithm to design DNA sequences that satisfied combinatorial constraints, including the Hamming distance constraint and accounting for GC content [12]. Shin used the Multi-objective evolutionary algorithm to design DNA sequences and developed a system (NACST) using a genetic algorithm [15], [16].

These studies describe a combination of distance constraint considerations and GC content to design DNA sequences with better thermodynamic properties; however, these methods only roughly constrain the thermodynamic attributes of DNA sequences. To address this gap, minimum free energy criteria are widely used to measure the thermodynamic properties of DNA sequences. Garzon and Rose proposed a method for measuring the quality of DNA sequence sets based on their thermodynamic properties by using statistical mechanic principles [17], [18], and Penchovsky and Ackermann employed combinatorial criteria to design sets of sequences for molecule-based computing [19]. To maximize the desired hybridization and minimize undesired hybridizations, they limited the range of the sequence set melting temperature. They proposed an important new ‘free energy gap’ measure of a set quality, and designed their sets based on this new constraint. Tulpan researched DNA sequence sets based on MFE with a PairFold package, which is freeware available online [9]. They described a new algorithm for designing DNA sequence sets in which sets would satisfy several thermodynamic and combinatorial distance constraints [7], [20]. This new technique aimed to maximize desired hybridizations between strands and their complements, while minimizing undesired false hybridizations. Garzon’s paper presents exhaustive research to produce DNA sequence sets of sizes comparable to maximal sets while guaranteeing the highest quality, as measured by the MFE between any pair of DNA sequences [21]. A comparison of their experimental results with previous work revealed improved lower bounds of DNA sequence sets based on MFE. Subsequently, Kawashimo [22] employed dynamic neighborhood searches to design DNA sequence sets and further improve Garzon’s methods. Kawashimo introduced a technique to reduce such time-consuming evaluations of MFE, rendering the dynamic neighborhood search strategy applicable to practical thermodynamic constraints. They increased the speed of local-search type algorithms for designing DNA sequence sets based on MFE [23] and their algorithm generated better DNA sequence sets than existing methods. Tulpan presented a quantitative comparison of four published DNA/DNA duplex free energy calculation methods and concluded that no statistically significant differences exist among free energy approximations in these publicly available and widely used programs. In another report, improved genetic algorithms were used to design DNA sequence sets which satisfied different combinational constraints and enabled the creation of the highest quality DNA sequences sets thus far [13]. Recently, Bystrykh proposed a method of generalized DNA barcode design based on Hamming codes [3]. In their work, Hamming barcodes could be employed for DNA tag designs in many different ways while preserving minimal distance and error-correcting properties. In the Xiao’s paper [24], a multi-swarm particle swarm optimization was proposed to deal with DNA encodings problem. The method proposed used the local PSO with the time-varying acceleration coefficients (TVAC) as the search engine for each sub-swarms, and incorporated the differential evolution to improve the swarm search space.

## Conclusions

Here we propose a novel distance constraint: the LACS distance for designing DNA sequences. This constraint decreases the similarity of DNA sequences and better models the thermodynamic properties of DNA in comparison to current Hamming distance constraints. The thermodynamic properties of different distances are accounted for using an improved genetic algorithm to design DNA sequence sets which satisfy the Hamming and LACS distances. Free energy gaps are used to gauge DNA sequence set quality. According to DNA sequence set sizes obtained using this improved genetic algorithm, we identified the lower bounds of novel constraints which satisfy different combinatorial constraints. Finally, we discussed the effect of different values of *l* and *k* on the free energy gaps of DNA sequence sets with identical DNA sequence lengths and distance constraints. We hypothesize that the maximal length of the LACS is even more important for designing DNA sequence sets based on thermodynamic properties.

Our work represents a valuable contribution to DNA sequence design. Future studies will improve our algorithm and the lower bounds based on the novel constraint. According to the proof based on Hamming distance, we could theoretically prove the exact lower and upper bounds of novel constraint and offer proof-of-concept for the theoretical relationship of *l* and *k*.
